# Comparative genomic landscape of lower-grade glioma and glioblastoma

**DOI:** 10.1371/journal.pone.0309536

**Published:** 2024-08-29

**Authors:** Xinxin Sun, Qingbin Jia, Kun Li, Conghui Tian, Lili Yi, Lili Yan, Juan Zheng, Xiaodong Jia, Mingliang Gu

**Affiliations:** 1 Joint Laboratory for Translational Medicine Research, Liaocheng People’s Hospital, Liaocheng, Shandong, China; 2 Department of Neurosurgery, Liaocheng People’s Hospital, Liaocheng, Shandong, China; Goethe University Hospital Frankfurt, GERMANY

## Abstract

Biomarkers for classifying and grading gliomas have been extensively explored, whereas populations in public databases were mostly Western/European. Based on public databases cannot accurately represent Chinese population. To identify molecular characteristics associated with clinical outcomes of lower-grade glioma (LGG) and glioblastoma (GBM) in the Chinese population, we performed whole-exome sequencing (WES) in 16 LGG and 35 GBM tumor tissues. *TP53* (36/51), *TERT* (31/51), *ATRX* (16/51), *EFGLAM* (14/51), and *IDH1* (13/51) were the most common genes harboring mutations. *IDH1* mutation (c.G395A; p.R132H) was significantly enriched in LGG, whereas *PCDHGA10* mutation (c.A265G; p.I89V) in GBM. *IDH1*-wildtype and *PCDHGA10* mutation were significantly related to poor prognosis. *IDH1* is an important biomarker in gliomas, whereas *PCDHGA10* mutation has not been reported to correlate with gliomas. Different copy number variations (CNVs) and oncogenic signaling pathways were identified between LGG and GBM. Differential genomic landscapes between LGG and GBM were revealed in the Chinese population, and *PCDHGA10*, for the first time, was identified as the prognostic factor of gliomas. Our results might provide a basis for molecular classification and identification of diagnostic biomarkers and even potential therapeutic targets for gliomas.

## Introduction

Gliomas are the most common malignant brain tumors in the central nervous system (CNS) [1]. Gliomas are classified into grades I to IV by the World Health Organization (WHO), mainly based on histology and malignancy [2]. Recently, molecular characteristics have been added as an important criterion in the revised classification system [3, 4]. The traditional treatment for glioma includes surgical resection, radiotherapy, and chemotherapy based on temozolomide (TMZ) [5]. These treatment leads to a better prognosis, especially grades II and III (LGG, with median survival time of more than 7 years) [6]. However, glioblastoma (GBM, WHO IV) still has poor prognosis (with the 5-year survival rate of 5.8%) [7].

Isocitrate dehydrogenase (IDH) mutations are one of the most critical molecular markers affecting the diagnosis, prognosis and treatment of gliomas. In gliomas, *IDH1* R132H is the most common mutation and the mutation is associated with slower progression and better prognosis. *IDH1* mutations occur in over 70% of LGG and GBM that progress from LGG and *IDH2* mutation occur in less than 5% in gliomas [8]. 2021 WHO classification systems of CNS includes three types of gliomas: *IDH*-mutant astrocytoma, *IDH*-mutant and 1p/19q-codeleted oligodendroglioma and *IDH*-wildtype GBM [4]. More and more molecular markers have been proven to play a crucial role in the classification, grading, prognosis, and treatment of gliomas. For example, *IDH*-wildtype astrocytic glioma carrying *TERT* promoter (*TERT*p) mutation, *EGFR* amplification, as well as gain-of-chromosome 7 and loss-of-chromosome 10 (+7/-10) are classified as GBM [4]. Although many studies have revealed the genomic landscape of gliomas based on Western/European populations predominantly, comparative genomic characteristics of LGG and GBM are yet to be displayed in Chinese patients [9–11]. Considering the impact of genetic backgrounds on the prognosis of gliomas and a lack of comparative genomics of LGG and GBM in the Chinese population, further comparison of the genomic characteristics between LGG and GBM in the Chinese population is essential. Here, we aim to analyze and compare genomic characteristics of 16 LGG and 35 GBM cases to explore both shared and grade-specific genomics of gliomas in Chinese patients, and thus offering new insight into potential molecular signatures and treatment targets.

## Materials and methods

### Selection of patients with gliomas and sample collection

Tumor tissues were obtained from 51 patients with primary gliomas from January 6, 2019 to July 1, 2020 in Liaocheng People’s Hospital and normal blood samples data was obtained from another study of our group [12]. These patients were histologically diagnosed with gliomas and underwent surgical resection. According to histopathological diagnosis, tumors were classified as grades II to IV. This study was approved by the medical ethics committee of Liaocheng People’s Hospital (No: 2019203, 6 January 2019) and strictly followed the guidelines of the Declaration of Helsinki. All involved patients had signed informed consent for participating in this study. Genomic DNA (gDNA) was extracted from fresh frozen tissues and peripheral blood samples using QIAamp DNA Mini Kit (Qiagen, Hilden, Germany). The quality and quantity of gDNA was analyzed by NanoDrop 1000 (Thermo Scientific, Wilmington, USA) and Qubit 3.0 Fluorometer (Life Technologies, Carlsbad, USA).

### Sanger sequencing and whole-exome sequencing

*IDH1* (codon R132), *IDH2* (codon R172) and *TERT promoter* (*TERT*p, C228T and C250T) mutations were detected by Sanger sequencing, using established primer (Table 1) [13, 14]. The primer sequences used for validation of *PCDHGA10* (codon I89) were listed in Table 1. DNA fragments were sequenced by Sangon Biotech (Sangon, Shanghai, China).

**Table 1:** Primer sequence of 4 genes.

Gene	Primer Sequence (5ʹ→3ʹ)
***IDH1***	Forward: CTCCTGATGAGAAGAGGGTTG
	Reverse: TGGAAATTTCTGGGCCATG
***IDH2***	Forward: TGGAACTATCCGGAACATCC
	Reverse: AGTCTGTGGCCTTGTACTGC
***TERT*p**	Forward: AGTGGATTCGCGGGCACAGA
	Reverse: GCAGCGCTGCCTGAAACTCG
***PCDHGA10***	Forward: AGGCTCTTTCGTGGGCAACATC
	Reverse: GCATTCCCGCAGCGACATTTTC

The whole exome was captured by SureSelect Human All Exon V6 Probes (Agilent, Santa Clara, USA) according to the manufacturer’s protocol. The qualified libraries were sequenced with Nextseq CN500 platform (Illumina, San Diego, USA) for 2×76 bp paired-end sequencing.

### Variant calling and annotation

Sequencing reads were mapped to human reference genome GRCh37/hg19 using Burrows-Wheeler Aligner (BWA) [15]. The duplicated reads were marked with Picard (http://broadinstitute.github.io/picard/) and realigned with Genome Analysis Toolkit (GATK) [16, 17]. All variants were annotated using ANNOVAR [18]. Variants were screened with 1000 Genomes Project (1KGP) [19], Exome Sequencing Project (ESP6500) (http://evs.gs.washington.edu/EVS/), The Exome Aggregation Consortium (ExAC) [20] and Catalogues of Somatic Mutations In Cancer (COSMIC) databases [21]. Variants were filtered out as follows: (1) variants presented in the 1KGP, ESP6500 or ExAC with a minor allele frequency of >1%; (2) base quality value of <20; (3) mutation reads depth of <10; (4) variant allele frequency (VAF) of < 5%; (5) synonymous variants.

### CNVs analysis

CNVkit was used to analysis CNVs with default parameter settings [22]. Peripheral blood-derived germline DNA samples of 30 healthy subjects were used as normal references for calculating tumor CNVs, which were generated using the same sequencing method and analysis strategy. CNVs were reported if the log2 (CN) was above 1.5 or below 0.5. Then, GISTIC2.0 was used to identify regions of significantly recurring gains or losses [23]. *q*-values were calculated from p-values of respective genomic regions determined by permutation test. Regions with a *q* < 0.25 were considered as significantly recurring gains or losses.

### Driver genes analysis

Cancer Gene Census (https://cancer.sanger.ac.uk/census/, CGC), Integrative Onco Genomics (https://www.intogen.org/search), BertVogelstein125 [24], SMG127 [25], and Comprehensive435 [26] were to identify known driver genes. Genes with high alteration frequencies identified at least one of the five data sources were considered as candidate driver genes [27].

### Enrichment analysis and associated cancer pathway analysis

Kyoto Encyclopedia of Genes and Genomes (KEGG) database (https://www.kegg.jp/kegg/) and Gene Ontology (GO) database were used for signaling pathway analysis and functional enrichment analysis, respectively [28, 29]. Ten oncogenic signaling pathways were analyzed as previously described, including TP53, WNT, MYC, HIPPO, PI3K, NOTCH, Cell Cycle, NRF2, TGFβ, and RTK/RAS pathways [30]. Patients having one or more mutations or CNV involved genes in these pathways were considered to have altered pathways [31]. Highlighted pathways were visualized by PathwayMapper tool (http://pathwaymapper.org) [32].

### Statistical analysis

Genetic mutations between LGG and GBM were compared with Fisher’s exact tests or Chi-square tests. 2-tailed *p* < 0.05 was considered significant. Kaplan-Meier estimate was illustrated for survival analysis, and log-rank test was used to determine the difference between two groups. The statistical analysis was performed using SPSS (Version23.0, Chicago, USA) or R v3.6.1 software.

## Results

### Clinical characteristics of patients with gliomas

Totally, 51 patients with gliomas, including 16 LGG and 35 GBM, were enrolled in this investigation (**Table 2 and S1 Table**). The median age was 51.41±13.39 years with a wide range (24–73 years). The majority of patients were male (n = 31, 60.78%). Most tumors were located in the frontal lobe (n = 17, 33.33%), temporal lobe (n = 17, 33.33%), followed by multiple regions (n = 15, 29.41%), parietal lobes (n = 1, 1.96%), and cerebellum (n = 1, 1.96%). *IDH1* mutation (c.G395A; p.R132H) was more common in LGG (n = 7, 43.75%) than GBM (n = 6, 17.14%). *TERT*p mutations (C228T and C250T) were more common in GBM (n = 22, 62.86%) than LGG (n = 9, 56.25%). Forty-seven out of 51 patients had available follow-up data. The median overall survival (OS) time of LGG and GBM cohort were 30.4 and 13.0 months, respectively.

**Table 2:** Clinical and genetic characteristics of 51 patients with gliomas.

Features	All (n = 51)	LGG (n = 16)	GBM (n = 35)
**Sex**			
**Male**	60.78% (31/51)	50.00% (8/16)	65.71% (23/35)
**Female**	39.22% (20/51)	50.00% (8/16)	34.29% (12/35)
**Age**			
**Median (Range)**	51.41 (24–73)	47.38 (24–64)	53.26 (24–73)
**Tumor location**			
**Frontal lobe**	33.33% (17/51)	37.50% (6/16)	31.43% (11/35)
**Temporal lobe**	33.33% (17/51)	25.00% (4/16)	37.14% (13/35)
**Two or more lobes**	29.41% (15/51)	37.50% (6/16)	25.71% (9/35)
**Other**	3.92% (2/51)	-	5.71% (2/35)
***IDH1***			
**Mutation**	25.49% (13/51)	43.75% (7/16)	17.14% (6/35)
**Wild-type**	74.51% (38/51) (38/51)	56.25% (9/16)	82.86% (29/35)
***TERT*p**			
**Mutation**	60.78% (31/51)	56.25% (9/16)	62.86% (22/35)
**Wild-type**	39.22% (20/51)	43.75% (7/16)	37.14% (13/35)
**Survival**			
**Yes**	23.40% (11/47)	60.00% (9/15)	6.25% (2/32)
**No**	76.60% (36/47)	40.00% (6/15)	93.75% (30/32)

### Mutational landscape of LGG and GBM patients

The average sequencing depth was 76.57× for 51 gliomas samples. 85.75% of target bases had a coverage of at least 30×. A total number of 5699 exonic mutations were detected in all patients, including 5330 missense mutations, 96 nonsense mutations, 57 frameshift Insertion–deletions (indels), 179 in-frame indels, and 37 unknown-function mutations (**S1A Fig**). Mutations with an unknown-function were removed, resulting in a median of 118 (range 96–134) variants, including 95.80% single nucleotide variants (SNV) and 4.20% indel mutations (**S1B Fig**). The major point mutations were C>T and T>C (**S1C Fig**).

**Fig 1:**
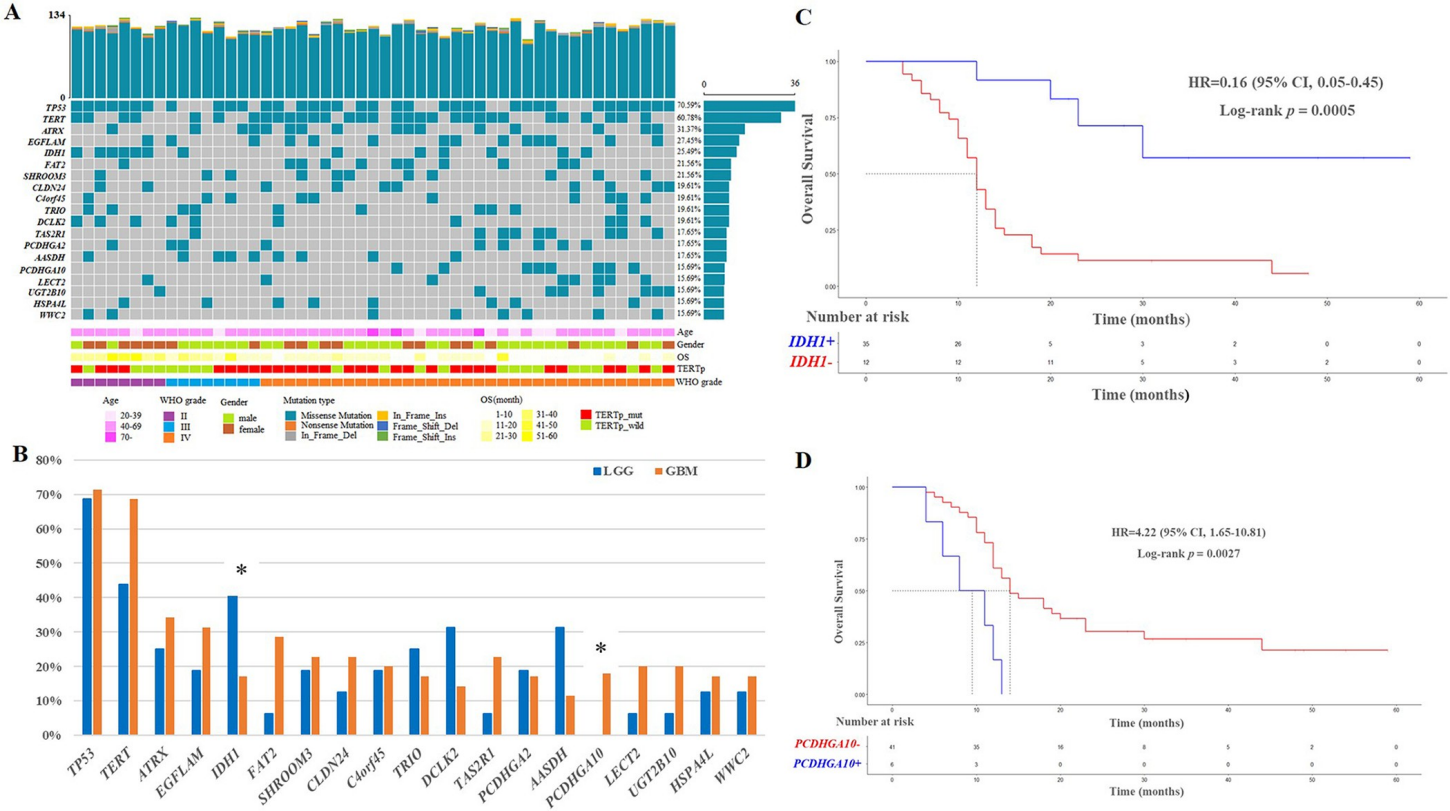
Recurrent genetic alterations in 51 gliomas. (A) Mutational landscape of gliomas. (B) Prevalence comparison of mutations in LGG and GBM (Chi-square test; *, *p* < 0.05). (C) OS of patients with *IDH1* mutation. (D) OS of patients with *PCDHGA10* mutation.

LGG and GBM shared 53 mutated genes, while 1800 and 3846 uniquely mutations were identified in LGG and GBM, respectively (**S1D Fig**). To demonstrate potential grade differences, those genes with a mutational frequency of > 15% were focused on. The top 5 mutated genes were *TP53* (n = 36, 70.59%), *TERT* (n = 31, 60.78%), *ATRX* (n = 16, 31.37%), *EFGLAM* (n = 14, 27.45%), and *IDH1* (n = 13, 25.49%) (**Fig 1A**). *TP53*, *ATRX* and *IDH1* were the candidate driver gene identified in all of the five data sources (**S2 Table**). The mutational rates were different between LGG and GBM. *IDH1*, *DCLK2*, and *AASDH* were enriched in LGG, while *FAT2*, *TAS2R1*, *PCDHGA10*, *LECT2*, and *UGT2B10* were enriched in GBM (**Fig 1B**). Mutations of *IDH1* and *PCDHGA10* were significantly different between LGG and GBM (*p* < 0.05, **Fig 1B**). *IDH1* mutation (c.G395A; p.R132H) was enriched in LGG (43.75% vs. 17.14%), whereas *PCDHGA10* mutation (c.A265G; p.I89V) in GBM (22.86% vs. 0%), which were consistent with Sanger sequencing (**S2 Fig**). Next, predictive values of *IDH1* and *PCDHGA10* mutations were analyzed. Patients with wild-type *IDH1* had shorter OS compared to those with mutated *IDH1* (14.29 months vs. 31.00 months, HR = 0.16; *p* = 0.0005; **Fig 1C**). Patients with mutated *PCDHGA10* had shorter OS compared to those with wild-type *PCDHGA10* (9.00 months vs. 19.95 months, HR = 4.22; *p* = 0.0027; **Fig 1D**).

Comparing with the public database TCGA and cBioPortal, significant differences were observed in mutational frequencies of various genes, such as *TERT*, *EGFLAM*, *IDH1*, *SHROOM3*, and *DCLK2* (**S3 Fig**).

**Fig 2:**
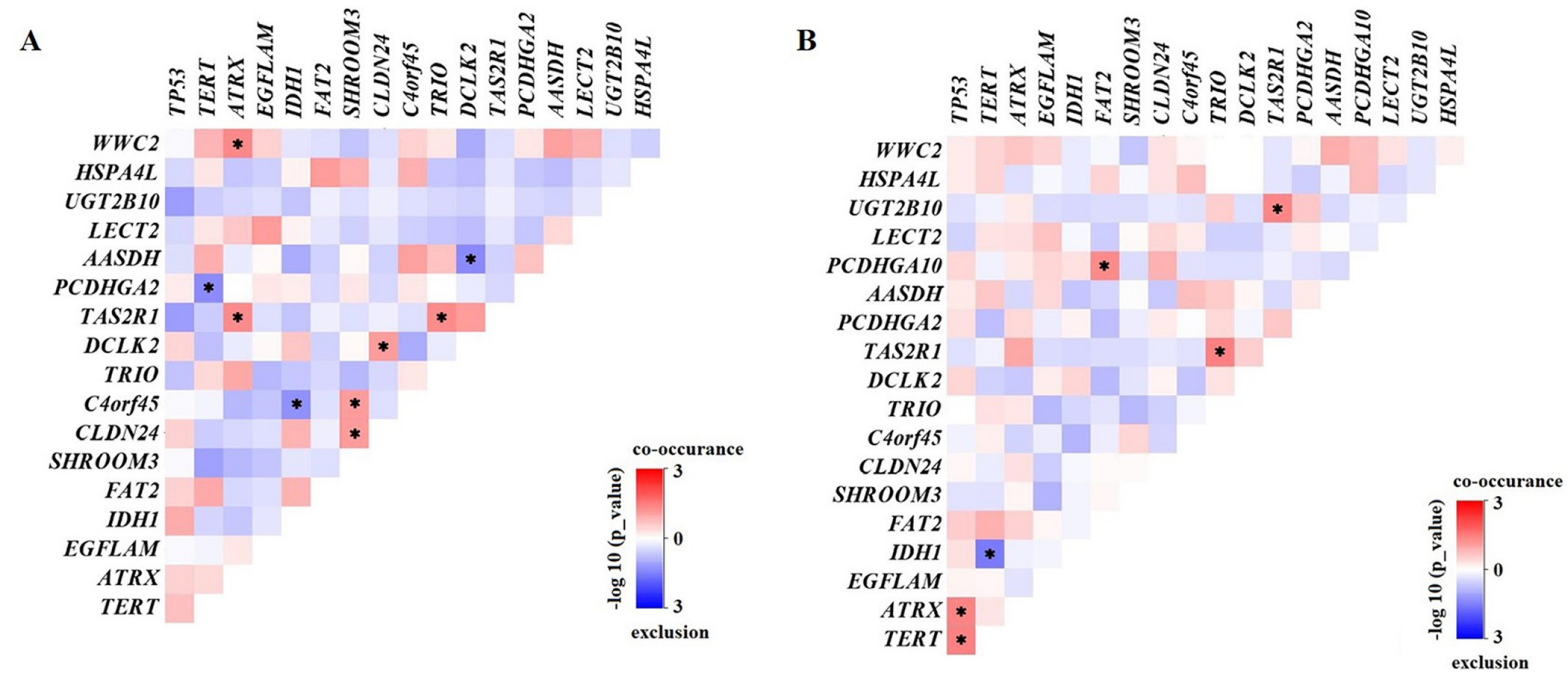
Concurrent and mutually exclusive somatic mutation patterns of mutated genes in LGG (A) and GBM (B). Significance was calculated using Fisher’s exact test, *p < 0.05.

Then, mutual coexistence and exclusion of 19 genes were analyzed by correlation analysis. Interacted gene pairs were significantly different between LGG and GBM. For example, interactions of *ATRX* with *WWC2* and *TAS2R1* were coexisted in LGG (**Fig 2A**). Interactions of *TP53* with *ATRX* and *TERT*, as well as interaction of *PCDHGA10* and *FAT2* were coexisted in GBM (**Fig 2B**).

### CNVs in LGG and GBM

**Fig 3:**
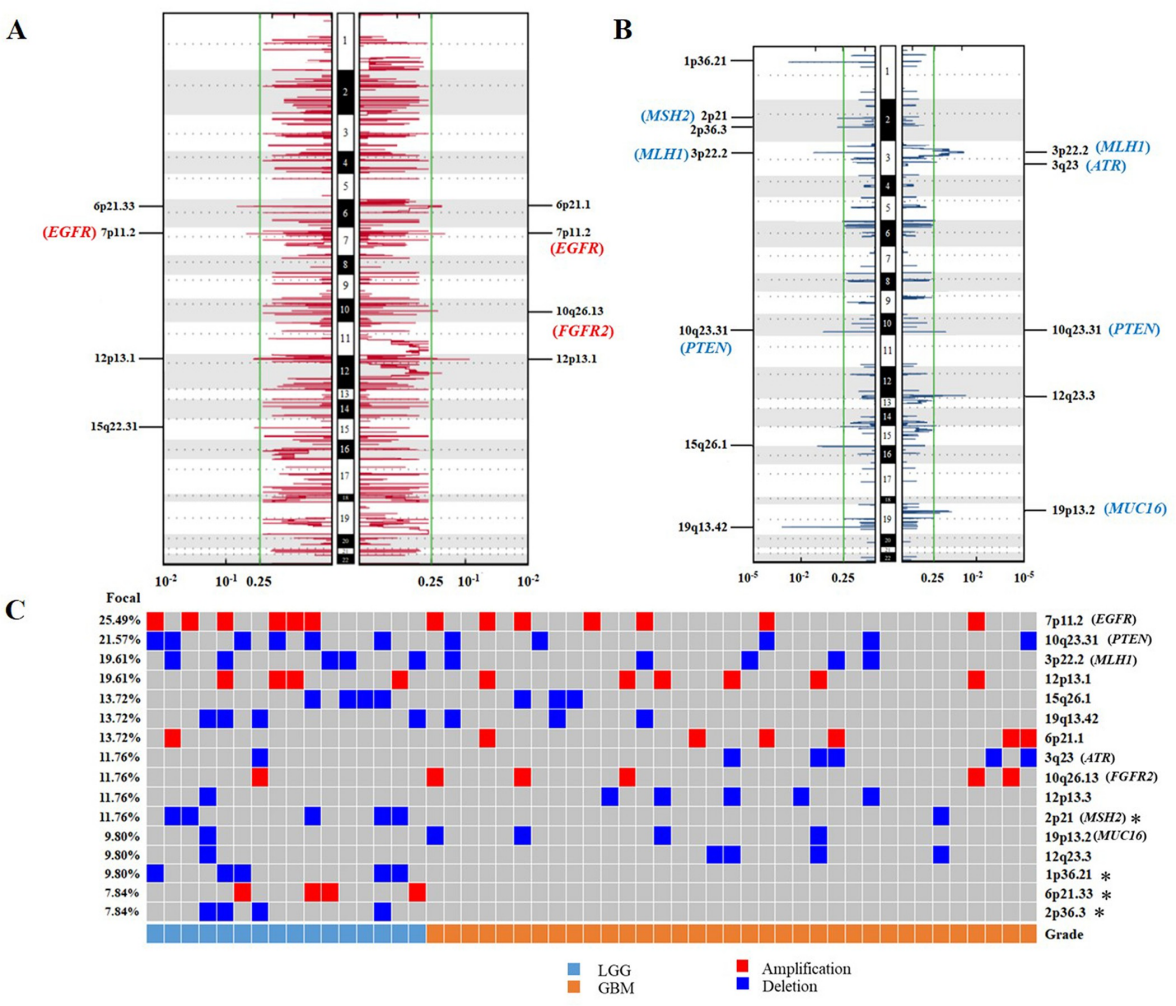
Differential CNVs between LGG and GBM. (A) Amplification regions in LGG (left) and GBM (right). (B) Deletion regions in LGG (left) and GBM (right). (C) Percentage of CNVs located in different chromosomal regions in LGG and GBM. The green line indicates the cut-off of significance (*q* = 0.25). Significance was calculated using Fisher’s exact test, *p < 0.05.

CNVs were analyzed by GISTIC2.0 in the LGG and GBM groups. We identified 6 significantly recurrent amplification regions (*q* < 0.25, **Fig 3A**) and 10 significantly recurrent deletion regions (*q* < 0.25, **Fig 3B**). Gain of chromosome 7 and loss of chromosome 10 were more common in gliomas (**Fig 3C**). In addition, according to potential driver genes in five data sources, we identified seven oncogenes or tumor-suppressor genes, including *EGFR* (7p11.2, 25.49%), *PTEN* (10q23.31, 21.57%), *MLH1* (3p22.2, 19.61%), *ATR* (3q23, 11.76%), *FGFR2* (10q26.13, 11.76%), *MSH2* (2p21, 11.76%), and *MUC16* (19p13.2, 9.50%) (**S2 Table** and **Fig 3C**). The LGG group had deletions in 2p21 (31.25% vs. 2.86%), 1p36.21 (31.25% vs. 0%), and 2p36.3 (25% vs. 0%), whereas amplification in 6p21.33 (25% vs. 0%) (*p* < 0.05, Fisher’s exact test, **Fig 3C**).

### Enrichment analysis and oncogenic signaling pathways

**Fig 4:**
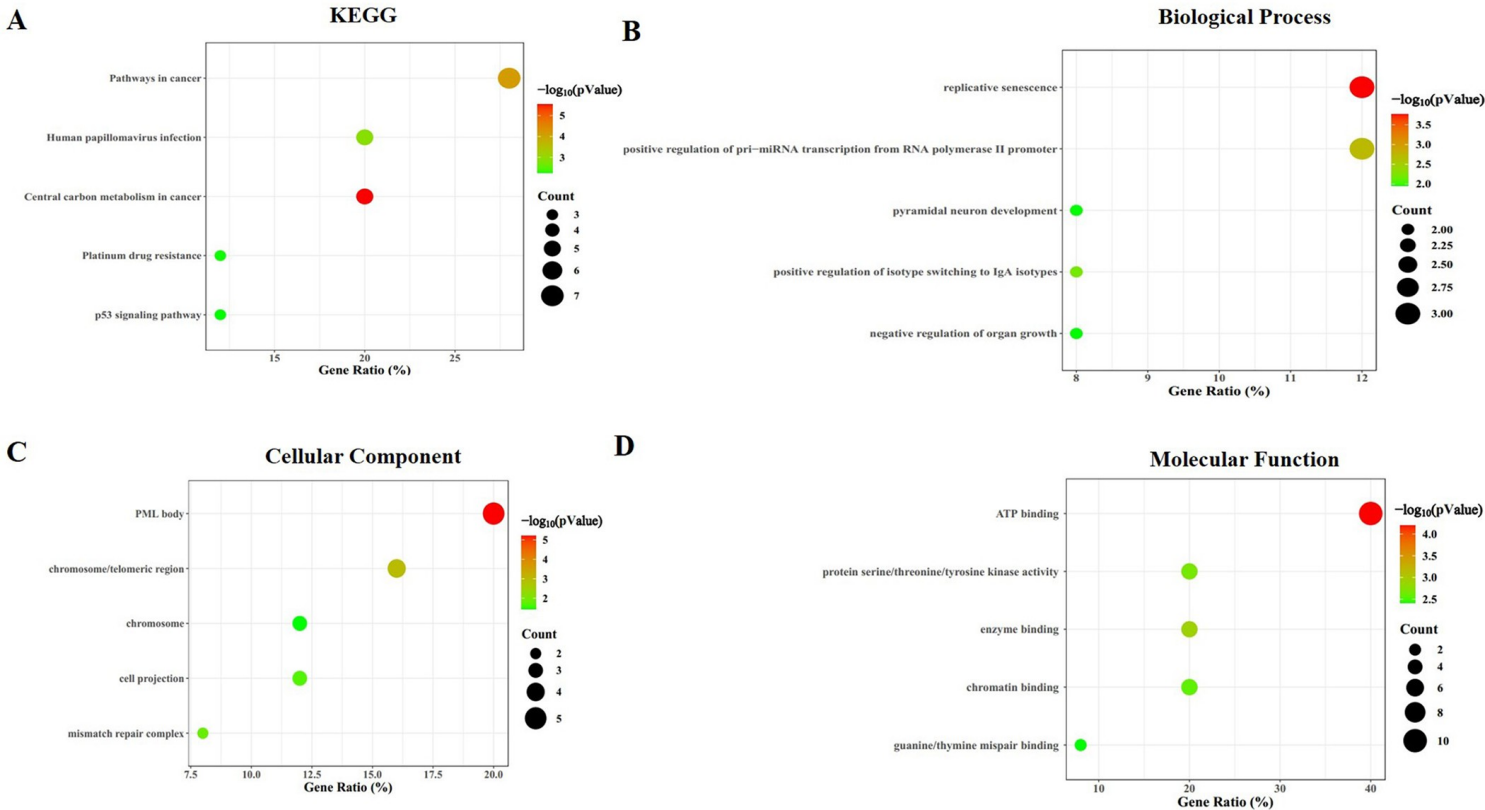
The significantly enriched KEGG pathways and GO annotations of frequently altered genes in gliomas cases. (A) KEGG pathway. (B) Biological processes. (C) Cellular components. (D) Molecular functions.

To understand the biological distributions of the frequently altered genes, we performed KEGG and GO analyses. KEGG analysis revealed that the frequently altered genes were highly enriched in “pathway in cancer” (**Fig 4A**). GO analysis indicated that mutated genes were mainly involved in replicative senescence and positive regulation of pri−miRNA transcription from RNA polymerase II promoter in biological processes (**Fig 4B**); PML body and chromosome/telomeric region in cellular components (**Fig 4C**) and ATP binding in molecular functions (**Fig 4D**).

**Fig 5:**
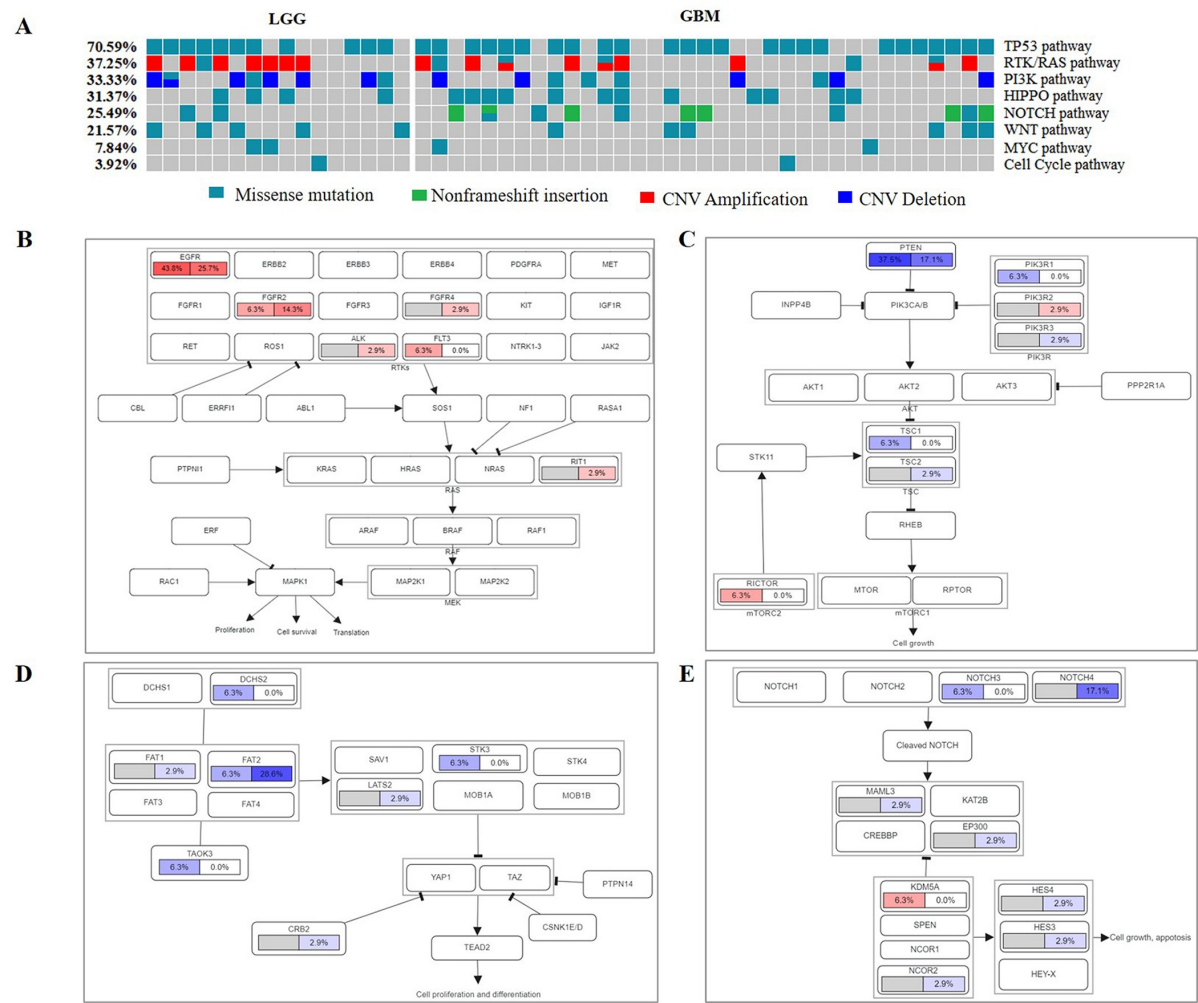
The frequencies of oncogenic signaling pathways altered in patients with gliomas. (A) The frequencies of oncogenic signaling pathways altered in gliomas. (B) RTK/RAS pathway. (C) PI3K pathway. (D) HIPPO pathway. (E) NOTCH pathway. Pathways were labeled with LGG on the left whereas GBM on the right. Red represents an oncogene while blue represents a tumor suppressor gene.

To compare oncogenic signaling pathways by genetic variations, we analyzed mutations and CNVs in LGG and GBM. Notably, TP53 (70.59%), RTK/RAS (37.25%), PI3K (33.33%), HIPPO (31.37%), and NOTCH (25.49%) pathways were frequently altered in gliomas. However, NRF2 and TGFB related genes were not found (**Fig 5A**). The most frequently mutated gene in the TP53 signaling pathway was *TP53*. There were differences in oncogenic signaling pathways between LGG and GBM. Interestingly, alterations in RTK/RAS (50% LGG vs. 31.43% GBM) and PI3K (50% LGG vs. 25.71% GBM) pathways preferentially occurred in LGG. Notably, alterations in HIPPO (34.29% GBM vs. 25% LGG) and NOTCH (28.57% GBM vs. 12.5% LGG) pathways were more common in GBM. RTK/RAS pathway was mainly caused by oncogene *EGFR* (43.8% LGG vs. 25.7% GBM, **Fig 5B**), while PI3K pathway activation was attributed to tumor suppressor gene *PTEN* (37.5% LGG vs. 17.1% GBM, **Fig 5C**). HIPPO pathway was modified by tumor suppressor gene *FAT2* (28.6% GBM vs. 6.3% LGG, **Fig 5D**), while NOTCH pathway was regulated by tumor suppressor gene *NOTCH4* (17.1% GBM vs. 0% LGG, **Fig 5E**).

## Discussion

In this study, we performed WES to compare molecular characteristics between 16 LGG with 35 GBM patients in the Chinese population. We have highlighted significant differences in genetic landscapes between LGG and GBM.

The most frequently mutated genes were *TP53*, *TERT*, *ATRX*, *EFGLAM*, and *IDH1* in 51 Chinese patients with gliomas. Except for *EFGLAM*, the other genes were biomarkers for gliomas [9, 33–35]. *EGFLAM* was related to poor prognosis in GBM as previously described [36]. Notably, mutations of *IDH1* and *PCDHGA10* were significantly different between LGG and GBM. *IDH1* mutation (c.G395A; p.R132H) was enriched in LGG and patients with *IDH1* mutation had better long-term survival. *IDH* mutations were identified in LGG and secondary GBM, as an important biomarker for longer OS in LGG [37]. By contrast, *PCDHGA10* mutation (c.A265G; p.I89V) was significantly enriched in GBM but not in LGG (22.86% vs. 0%). In addition, *PCDHGA10* mutation correlated with shorter OS time. *PCDHGA10* might be a potential biomarker for prognosis in gliomas. *PCDHGA10* mutation (c.A265G; p.I89V) has not been reported to be associated with gliomas, which has been reported in other tumors (bladder cancer, gastric adenomas, and gastrointestinal stromal tumors) [38–40]. *PCDHGA10* other mutations (such as c.G1765A, p.G589S; c.A395G/T, D132G/V) have been reported in astrocytoma grade IV [41, 42]. *PCDHGA10* mutational frequency was low in the TCGA GBM (0.8%, 3/374) and cBioPortal GBM (1.01%, 4/397). *PCDHGA10* is a member of *Pcdh-γ* gene clusters. There has been accumulating evidence that members of PCDH family act as tumor suppressor genes in several types of cancer [43–47]. For example, knockdown of PCDHGA9 promoted migration and invasion of gastric cancer cells, while PCDHGA9 overexpression inhibited proliferation and metastasis of gastric cancer cells [43]. PCDHGA10 is significantly upregulated in lung squamous cell carcinoma (LUSC) and a high level of PCDHGA10 expression is associated with a poorer prognosis. PCDHGA10 might be a potential molecular marker in LUSC [48]. However, the potential role of *PCDHGA10* in tumorigenesis of gliomas has not been reported. Therefore, further studies are required to investigate the pathogenic functions of PCDHGA10 in GBM, especially in Chinese population.

Using CNVs analysis, 6 amplification regions and 10 deletion regions were defined as significantly recurrent CNVs. Oncogenes *EGFR*, *FGFR2* and *MUC16*, as well as tumor-suppressor genes *PTEN*, *MLH1*, *ATR*, and *MSH2* were identified according to driver genes in five data sources. *EGFR* amplification and *PTEN* deletion have values in diagnosis, response to therapy and prognosis in molecular subgroups of gliomas [49]. *EGFR* amplification occurred in 40–60% of GBM, which may serve as an attractive therapeutic target in GBM [50]. Approximately 70% of GBM had *PTEN* loss [51], however, *PTEN* loss as a prognostic factor has not been verified and remains controversial [52]. *EGFR* and *PTEN* could alter receptor tyrosine kinase (RTK)/PI3K/AKT/mTOR pathway and promote tumor progression [53]. In our study, LGG had deletions in 1p36.21, 2p21 and 2p36.3, whereas amplification in 6p21.33. Notably, 1p36.21 deletion was related to poor prognosis in astrocytoma and neuroblastoma [54, 55].

Enrichment analysis revealed that the frequently altered genes were involved in complex pathways in cancer (e.g., TP53, RTK/RAS and PI3K pathway), biological processes (e.g., replicative senescence and positive regulation of pri−miRNA transcription), cellular components (e.g., ML body and chromosome/telomeric region), and molecular functions (e.g., ATP binding and enzyme binding). In patients with gliomas, the most common mutations were harbored in P53 (70.59%), RTK/RAS (37.25%), PI3K (33.33%), HIPPO (31.37%) and NOTCH (25.49%) pathways. 78% of GBM had alterations in the p53 pathway [51], which might promote LGG progression to GBM [56]. Dysfunctional RTK/RAS pathway was attributed to EGFR, whereas PI3K pathway attributed to *PTEN*. Mutated *EGFR* and *PTEN* activate PI3K pathway, leading to activation of downstream AKT/mTOR cascade [57]. AKT/mTOR pathway promotes proliferation, survival and migration [58]. Dysregulated HIPPO pathway was mainly caused by mutated *FAT2*. *FAT2* may be involved in cancer aggressiveness, which remains inconclusive [59]. Pathogenic NOTCH pathway may regulate tumor initiation, progression, and recurrence of gliomas. However, the role of NOTCH pathway in gliomas development is debated [60].

In our study, genomic profiles of gliomas were similar to public databases like TCGA and cBioPortal, such as *TP53*, *IDH1*, *ATRX*, *EGFR* and *PTEN*. However, most of gene mutation frequencies were significant differences between our cohort and public databases. Due to different human lineages have genetic heterogeneity, which was shaped by many factors, including evolutionary history, environmental exposures, and lifestyle practices. In addition, the sample size of our cohort was relatively small, which may cause uncertainty frequency counting of gene alterations.

Several limitations should be mentioned in this study. First, the sample size of this retrospective cohort was relatively small. Furthermore, this study was based on single omics, which might lack adequate validation information. In addition, *PCDHGA10* might be related to the prognosis of GBM, however, additional experiments are required to explore pathologic functions of *PCDHGA10* in gliomas. Hence, we should conduct multi-center studies with larger sample sizes to validate our findings with multi-omics platforms.

## Conclusion

This study reveals comprehensive genomic characteristics of LGG and GBM in Chinese patients, which may provide a better understanding of differential molecular signatures between LGG and GBM. In addition, *PCDHGA10* mutation might be a novel biomarker for poor prognosis in GBM. The discovery of unique molecular biomarkers could contribute to glioma classification, help predict prognosis and provide therapeutic options. These results need to be confirmed by comprehensive studies with larger sample sizes, especially for a prognostic role of *PCDGHA10* in GBM.

## Supporting information

S1 Fig The analysis of WES in 16 LGG and 35 GBM patients. (A) Number of each type of mutation. (B) Number of SNV and Indels in all mutations. (C) Distribution of point mutation types in SNV. (D) The Venn diagram showed the number of co-mutated and uniquely-mutated genes in LGG and GBM patients.
(TIF)

S2 Fig The mutation of the *IDH1* and *PCDHGA10*. (A) Hot spots of mutations in the *IDH1* gene. (B) Sanger sequencing results of the novel mutation of the *IDH1* gene. (C) Hot spots of mutations in the *PCDHGA10* gene. (D) Sanger sequencing results of the novel mutation of the *PCDHGA10* gene.
(TIF)

S3 Fig Comparison of mutation frequencies between our study and cBioPortal database (A) and TCGA database (B).
(TIF)

S1 Table Clinical data of all gliomas. (XLSX)

S2 Table Identified driver genes. (XLSX)
