# Supplementary figures and images for: Comparative genomic landscape of lower-grade glioma and glioblastoma
Source: PLoS One. 2024 Aug 29;19(8):e0309536. doi: 10.1371/journal.pone.0309536 (PMC11361568; doi:10.1371/journal.pone.0309536)

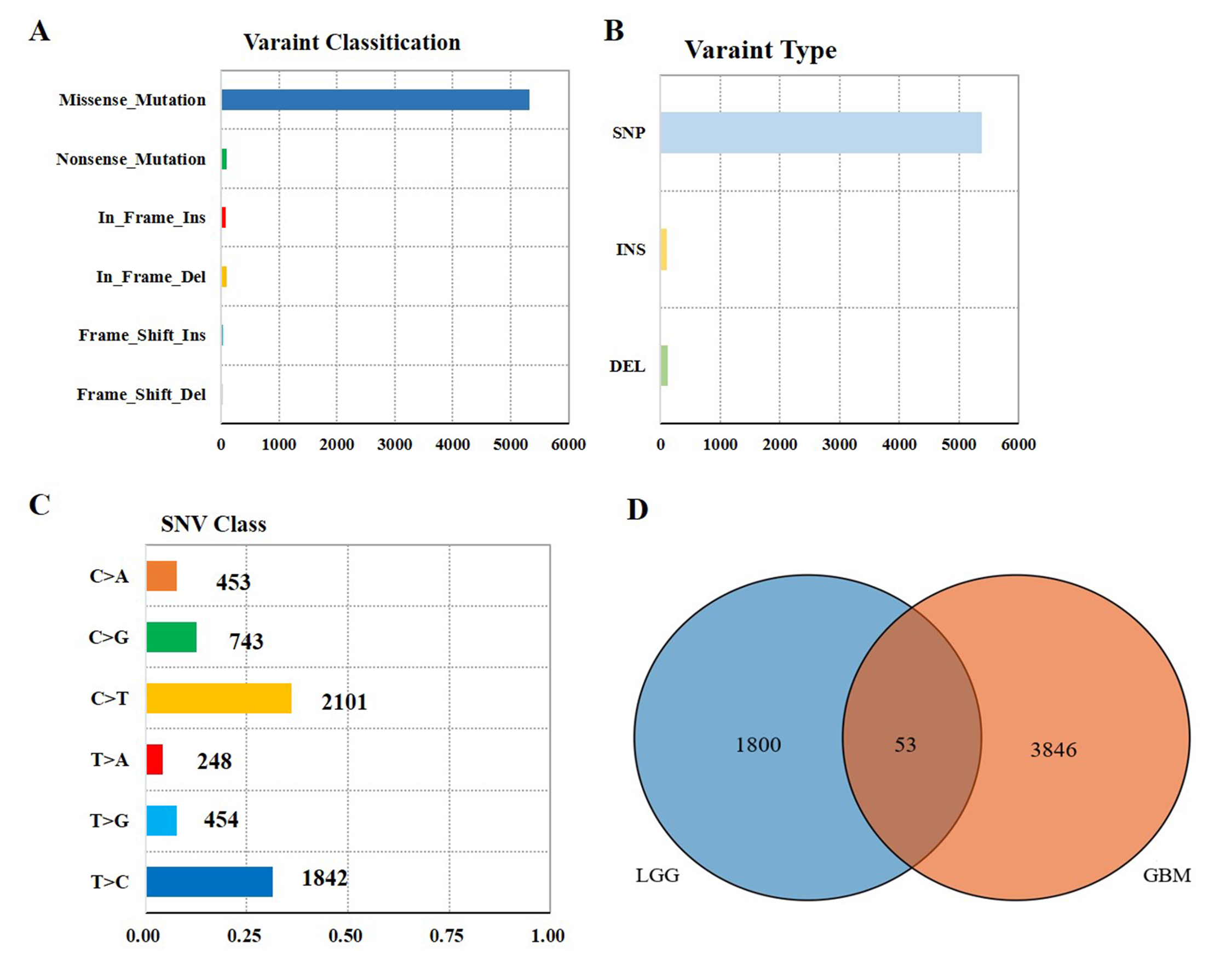

Supplement: S1 Fig — (A) Number of each type of mutation. (B) Number of SNV and Indels in all mutations. (C) Distribution of point mutation types in SNV. (D) The Venn diagram showed the number of co-mutated and uniquely-mutated genes in LGG and GBM patients. (TIF) [file pone.0309536.s001.tif]

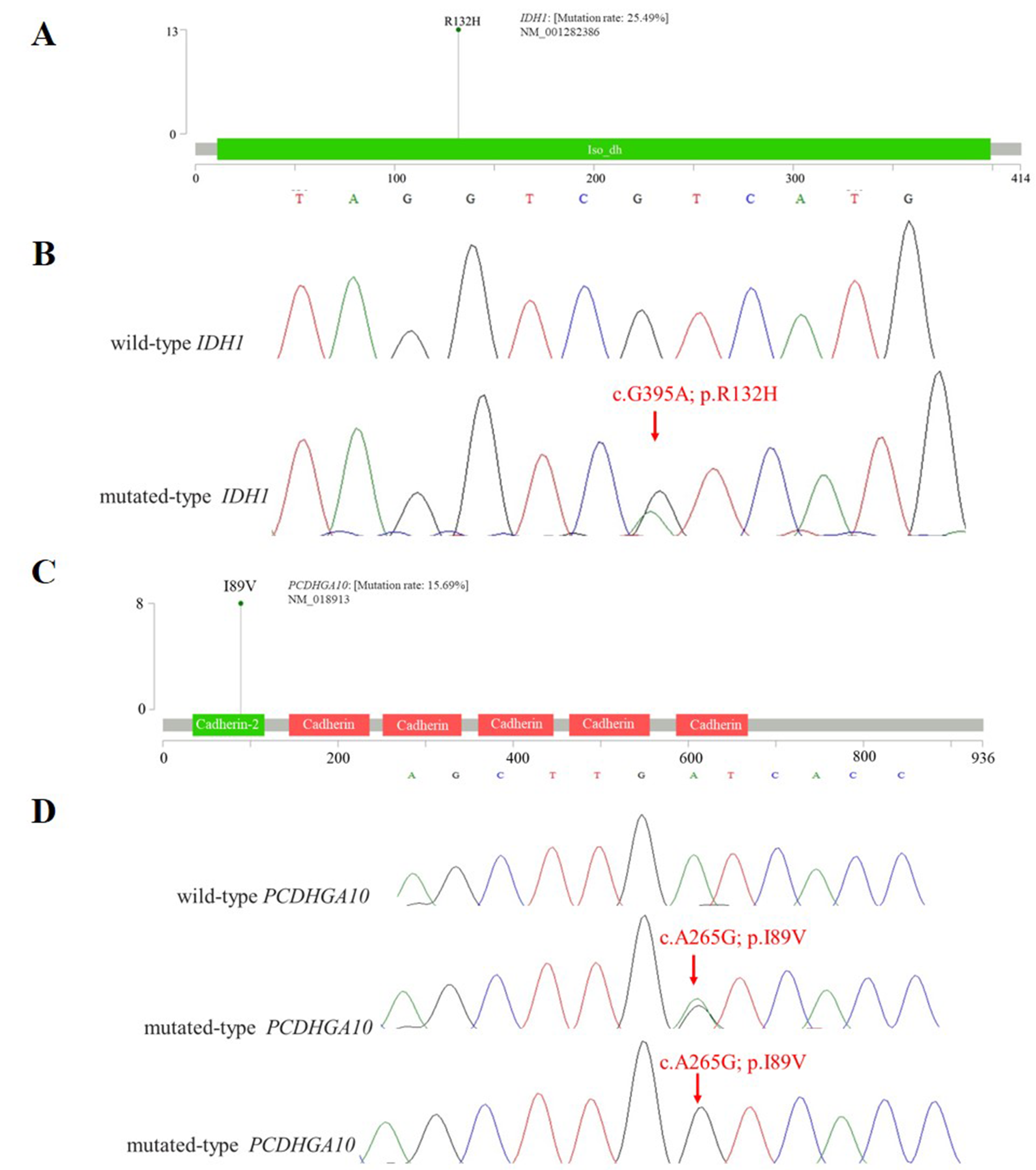

Supplement: S2 Fig — (A) Hot spots of mutations in the IDH1 gene. (B) Sanger sequencing results of the novel mutation of the IDH1 gene. (C) Hot spots of mutations in the PCDHGA10 gene. (D) Sanger sequencing results of the novel mutation of the PCDHGA10 gene. (TIF) [file pone.0309536.s002.tif]

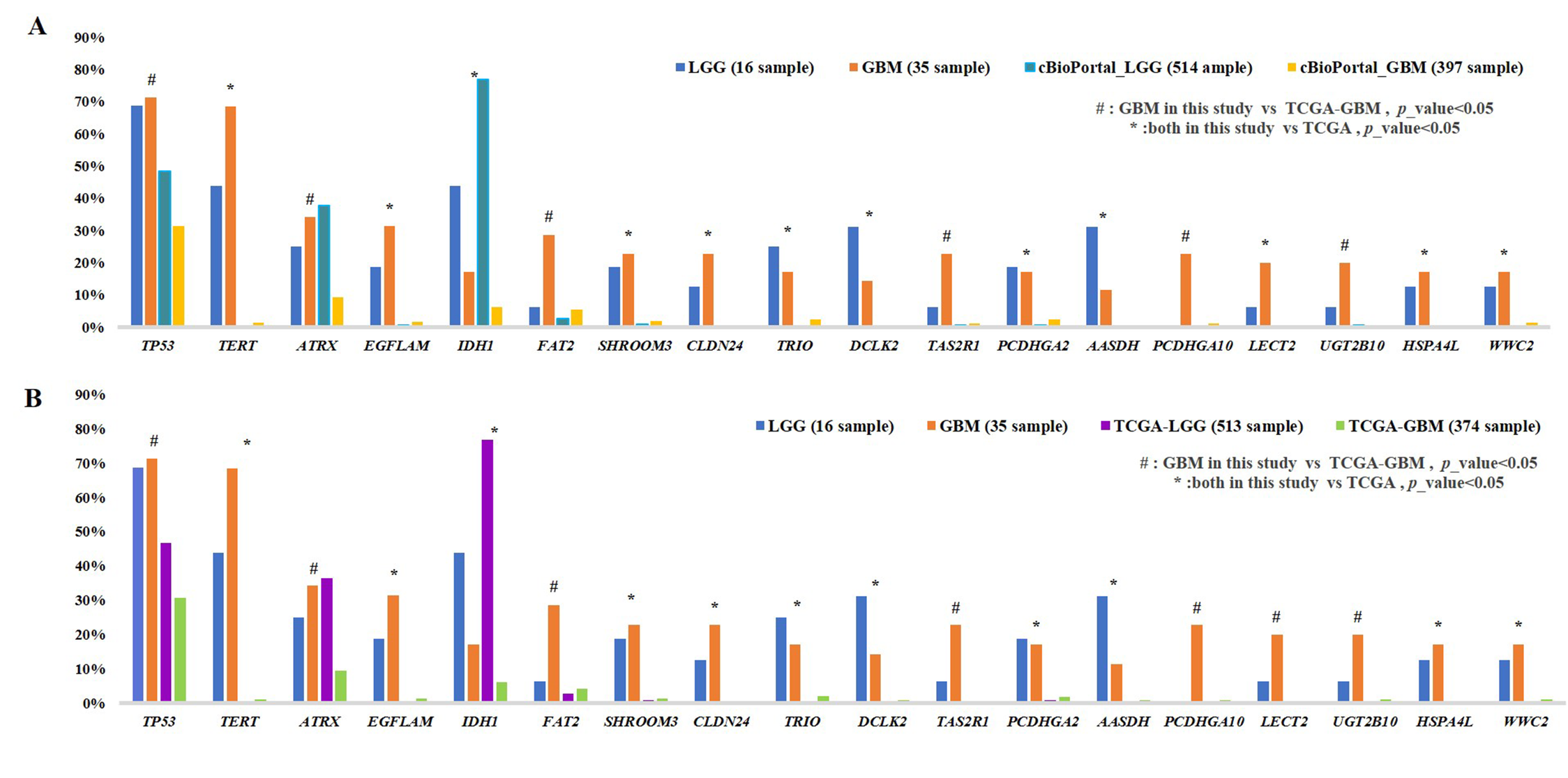

Supplement: S3 Fig — Comparison of mutation frequencies between our study and cBioPortal database (A) and TCGA database (B). (TIF) [file pone.0309536.s003.tif]
